# Clustering Analysis of the Multi-Microbial Consortium by *Lactobacillus* Species Against Vaginal Dysbiosis Among Ecuadorian Women

**DOI:** 10.3389/fcimb.2022.863208

**Published:** 2022-05-11

**Authors:** David Pacha-Herrera, Maria P. Erazo-Garcia, Darío F. Cueva, Miguel Orellana, Pamela Borja-Serrano, Camila Arboleda, Eduardo Tejera, António Machado

**Affiliations:** ^1^ Universidad San Francisco de Quito USFQ, Colegio de Ciencias Biológicas y Ambientales COCIBA, Instituto de Microbiología, Laboratorio de Bacteriología, Quito, Ecuador; ^2^ Facultad de Ingeniería y Ciencias Agropecuarias, Grupo de Bioquimioinformática, Universidad de Las Américas, Quito, Ecuador

**Keywords:** hierarchical clustering analysis, *Lactobacillus* species, vaginal microbiota, bacterial vaginosis, aerobic vaginitis

## Abstract

The vaginal microbiota plays vital protection in women. This probiotic activity is caused not only by individual *Lactobacillus* species but also by its multi-microbial interaction. However, the probiotic activity promoted by multi-microbial consortia is still unknown. The aim of this study was the individual and collective analysis on the prevalence of five vaginal lactobacilli (*Lactobacillus iners*, *Lactobacillus crispatus*, *Lactobacillus gasseri*, *Lactobacillus jensenii*, and *Lactobacillus acidophilus*) among healthy women and women with bacterial vaginosis (BV) or aerobic vaginitis (AV). PCR assays were realized on 436 vaginal samples from a previous study. Chi-square, univariable, and multivariable logistic regression analyses with the Benjamini–Hochberg adjustment evaluated associations between these lactobacilli and vaginal microbiota. Multi-microbial clustering model was also realized through Ward’s Minimum Variance Clustering Method with Euclidean squared distance for hierarchical clustering to determine the probiotic relationship between lactobacilli and vaginal dysbiosis. Concerning the individual effect, *L. acidophilus*, *L. jensenii*, and *L. crispatus* showed the highest normalized importance values against vaginal dysbiosis (100%, 79.3%, and 74.8%, respectively). However, only *L. acidophilus* and *L. jensenii* exhibited statistical values (*p* = 0.035 and *p* = 0.050, respectively). *L. acidophilus* showed a significant prevalence on healthy microbiota against both dysbioses (BV, *p* = 0.041; and AV, *p* = 0.045). *L. jensenii* only demonstrated significant protection against AV (*p* = 0.012). Finally, our results evidenced a strong multi-microbial consortium by *L. iners*, *L. jensenii*, *L. gasseri*, and *L. acidophilus* against AV (*p* = 0.020) and BV (*p* = 0.009), lacking protection in the absence of *L. gasseri* and *L. acidophilus*.

## Introduction

Vaginal microbiota balances the health state of women through its ability to prevent potential dysbiosis or infections (Pacha-Herrera et al., 2020; Joseph et al., 2021). Healthy women usually show a diversity of anaerobic and aerobic microorganisms in the vaginal epithelium (Borges et al., 2013), in which lactobacilli are the dominant species and act as a protective barrier to prevent pathogenic colonization (Di Cerbo et al., 2016; Scillato et al., 2021). However, the vaginal colonization by different lactobacilli species depends also on their ability to produce antimicrobial compounds, such as hydrogen peroxide, lactic acid, and bacteriocin-like substances (Borges et al., 2013; Castillo-Juárez et al., 2022). These antimicrobial compounds are extremely important in the impairment of colonization by pathogens associated with different types of vaginitis or dysbiosis, such as bacterial vaginosis (BV), vulvovaginal candidiasis (VC), and aerobic vaginitis (AV) (Vaneechoutte, 2017b; Vaneechoutte, 2017a). Vaginal dysbiosis increases public health costs and affects women of reproductive age who will develop chronic infections and more serious outcomes (Van De Wijgert et al., 2014; Walker, 2016), such as infertility, miscarriage, chronic pelvic inflammation, and an augmented HIV transmission (Onderdonk et al., 2016; Oostrum et al., 2018).

Different *Lactobacillus* species are usually found in the vaginal microbiota of healthy women, such as *Lactobacillus iners*, *Lactobacillus crispatus*, and *Lactobacillus gasseri* (Cribby et al., 2008; Vaneechoutte, 2017b). Despite that *L. iners* is found in the vaginal microbiota of healthy women, this bacterial species is also associated with transient or BV-associated microbiota, as previously discussed (Petrova et al., 2017). It is also well-known that significant differences in lactobacilli composition on the vaginal tract among women of different countries, races, and ethnicities are commonly found (Zhou et al., 2007; Madhivanan et al., 2014; Van De Wijgert et al., 2014; Borgdorff et al., 2017). Likewise, variations on microbial consortia among women with different vaginitis or dysbiosis are frequently reported (Demba et al., 2005; Borgdorff et al., 2017). However, most studies on Latin American mainly focus on determining BV prevalence (Kenyon et al., 2013; Krauss-Silva et al., 2014), and little is still known about the lactobacilli composition and their prevalence in Latin American women (Salinas et al., 2018; Peebles et al., 2019). Therefore, our main goal is to characterize the prevalence of five well-known lactobacilli species (*L. iners*, *L. crispatus*, *L. gasseri*, *Lactobacillus jensenii*, and *Lactobacillus acidophilus*) in the vaginal microbiota of native Ecuadorian women from our previous epidemiological study (Salinas et al., 2020). The present study assessed the presence of these lactobacilli using PCR amplification of *16S* and *23S* rRNA genes, and further multiple comparisons evaluated potential correlations between *Lactobacillus* species and sociodemographic factors and different types of vaginal microbiota (healthy microbiota, intermediate microbiota, and vaginal dysbioses, such as BV and AV) through chi-square, univariable, and multivariable logistic regression analyses with the Benjamini–Hochberg (BH) adjustment. Finally, a multi-microbial clustering model was also realized through Ward’s Minimum Variance Clustering Method with Euclidean squared distance for hierarchical clustering fed to determine any potential symbiotic or antagonistic relationship between these *Lactobacillus* species against both cases of vaginal dysbiosis.

## Materials and Methods

### Study Design

This study was conducted in the Microbiology Institute at the Universidad San Francisco de Quito (USFQ) from June 2017 to November 2018. As previously reported (Salinas et al., 2020), 436 Ecuadorian women of Hispanic ethnicity between 18 and 56 years old volunteered to be part of the epidemiological study. Briefly, all women received a kit containing an informed consent approved by the Bioethics Committee of the USFQ, a standardized medical survey, and a vaginal transport swab system (Stuart’s transport media swabs; Copan Diagnostics Inc., Brescia, Italy). Volunteers were excluded if they reported having had sexual intercourse within the last 48 h, antimicrobial treatment in the last 3 months, or any evidence of bleeding. The study was supervised by a physician, a psychologist, and a full-time researcher from the USFQ. This investigation adopted a cross-sectional study design to determine the association between the presence of five well-known lactobacilli species and vaginal microbiota or opportunistic pathogens (such as *Gardnerella* spp., *Fannyhessea vaginae* previously known as *Atopobium vaginae*, *Mobiluncus* spp., *Escherichia coli*, and *Candida albicans*) previously diagnosed/detected in our last publication (Salinas et al., 2020), more exactly, healthy microbiota, intermediate microbiota, and vaginal dysbioses (AV and BV, and VC).

### Ethics Statement

The present study was approved by the Ethics Committee of the USFQ (Protocol code: 2016-023IN by MSP-VGVS-2016-0244-O review board).

### DNA Extraction

DNA extraction was realized through standard procedure following Peng and colleagues’ direct boiling point method (Peng et al., 2013). Briefly, the stored aliquots (0.9% NaCl) of 1 ml were incubated at 100°C in a water bath for 15 min and then immediately frozen at −20°C for 15 min. Next, the samples were centrifuged at 13,000 rpm for 15 min, and supernatants were aliquoted into 500-μl volumes. DNA quantification was performed with a NanoVue spectrophotometer (GE Healthcare Life Science, Marlborough, MA, USA), samples were eluted at 20 ng/µl with molecular grade water and stored at −20°C until the PCR analysis was performed. The quality of DNA was evaluated by measuring the concentration of phenolic compounds or the presence of salts (260/230) and protein contaminants (260/280). This procedure was adapted from Money (Money, 2005).

### Identification of *Lactobacillus* Species by PCR

From our previous study (Salinas et al., 2020), 436 vaginal samples were selected for molecular characterization by PCR in a Bio-Rad Thermocycler (Bio-Rad, Hercules, CA, USA). DNA quantification was performed with a NanoVue spectrophotometer (GE Healthcare Life Science) to ensure the presence of amplifiable DNA. Concentrations of DNA in ng/μl were measured, as well as the phenolic contaminants (260/230) and the protein contaminants (260/280). Aliquots of DNA between 10 and 20 ng/µl were used for PCR analysis. Before lactobacilli detection was realized, all samples were analyzed for *16S* conserved rRNA genes (fDD2-CCGGATCCGTCGACAGAGTTTGATCITGGCTCAG; rPP2-CCAAGCTTCTAGACGGITACCTTGTTACGACTT) by PCR, ensuring the absence of PCR inhibitors on samples, as previously described (Borja-Serrano et al., 2020). All samples were analyzed with a total of five primer pairs, targeting five *Lactobacillus* species (*L. acidophilus*, *L. crispatus*, *L. gasseri*, *L. jensenii*, and *L. iners*). Single-template PCR assays were performed for each primer set. The sequence, amplicon size, target species, and temperature of annealing for each primer pair are described in **Supplementary Table 1**. A final volume of 20 µl was used according to the reference protocols (Galán et al., 2006; Fredricks et al., 2007; Sepehri et al., 2009; Henriques et al., 2012; DTU- National Food Institute, 2014), which included 0.5 U of Go Taq^®^ DNA Polymerase (Promega, Madison, WI, USA), 1× of Green GoTaq^®^ Flexi Buffer (Promega), 0.25 mM of MgCl_2_ (Promega), 200 µM of dNTP mix (Promega), 0.5 µM of each primer and target template DNA concentration of approximately 4 ng/μl, and the remaining volume with molecular grade H_2_O. The PCR thermal cycling consisted of initial denaturation at 94°C for 2 min, followed by 29 cycles of denaturation at 94°C for 30 s, annealing at each primer pair temperature for 30 s and extension at 72°C for 1 min, and final extension of 5 min at 72°C. The respective use of negative (without DNA sample and samples with other related bacteria) and positive (collection of identified strains of each species through DNA sequencing) controls were used in each PCR assay. These positive controls were provided by the Microbiology Institute at USFQ. All samples were randomly performed in triplicate with different negative and positive controls. After PCR amplification, a volume of 4 µl from each PCR product was visualized in 1.5% (w/w) agarose (Promega) gel electrophoresis using 0.1% ethidium bromide staining. The DNA analysis was performed under permit No. MAE-DNB-CM-2016-0046 (De Backer et al., 2007; Garg et al., 2009; Tsai et al., 2010; Zhang et al., 2012).

### Statistical Analysis

A multivariate logistic regression model was used to calculate the odds ratios (OR) of the clinical outcomes that included demographic variables (age, sex, city, and marital status), socioeconomic variables (occupation and level of education), personal habits (sex relationships, hygiene, and other habits), and the type or number of vaginal *Lactobacillus* species associated with the presence or absence of vaginal infection using logistic regression. These data were also considered categorical variables. Firstly, the variable of vaginal infection in the samples was categorized as the presence and absence, so a comparison of the different risk factors of both groups can be performed. After further statistical analysis, the study was defined by the type of vaginal dysbiosis (BV and AV) for testing differences in the previously analyzed factors and vaginal microbiota. The chi-square test was used to evaluate associations between the prevalence of vaginitis with the other risk factors. A value of *p* < 0.05 and 95% CIs were considered significant for the test. Logistic regression was also performed to calculate crude ORs for each variable mentioned; adjusted ORs were produced for variables with statistical significance in both tests applied for association (Ozaydin et al., 2013; Porras et al., 2014; Syam et al., 2015). Therefore, the chi-square test was used as a test of association, while the OR was then used as a measure of association (Kim, 2017). The statistical analysis of association with risk factors was performed for each type of vaginal infection but negative for the remaining types of vaginal infection to observe a significant difference between those populations. Each type of vaginal infection, normal or healthy microbiota, and intermediate microbiota were classified as dependent variables against sociodemographic and behavioral variables or the presence of *Lactobacillus* species as independent variables. All initial values of *p* < 0.05 obtained by univariable logistic regression, chi-square, and multivariable logistic regression analyses were then evaluated through the BH adjustment to detect false discovery rate (FDR) for conducting multiple comparisons. All statistical analyses were performed using SPSS version 22.0 (SPSS Statistics for Windows Version 22.0, IBM Corp, Armonk, NY, USA), except for the BH adjustment. The BH adjustment was realized using Seed-based d Mapping software (SDM, version 6.21, https://www.sdmproject.com, formerly “Signed Differential Mapping”) (Radua and Mataix-Cols, 2009; Radua et al., 2012). A clustering model was realized through Ward’s Minimum Variance Clustering Method with Euclidean squared distance to perform hierarchical clustering fed by a dimensionality reduction algorithm Principal Component Analysis (PCA) implemented in RStudio software (version 1.3.1073; https://rstudio.com/), using the option method = “ward” of the hclust function from the stats base R package (Package stats version 4.1.0) (Murtagh and Legendre, 2014).

## Results

### Description of Study Population

A total of 436 women volunteered in our last study (Salinas et al., 2020), with their vaginal samples and epidemiologic data selected for lactobacilli characterization in the present study. The stored samples were chosen for the molecular analysis by PCR. As shown in **Table 1**, our population set was constituted by Ecuadorian women between 18 and 56 years old, with 76.3% between 18 and 28 years old. Briefly, 66.1% of the women have healthy vaginal microbiota, 10.8% have an intermediate microbiota, and finally, 23.1% showed vaginal dysbiosis or infections. Among women with vaginal dysbiosis or infection, AV was the main vaginal dysbiosis being diagnosed in 52.5% (53/101), followed by BV (23.8%; 24/101) and VC (6.9%; 7/101). Eighty-four women were diagnosed with a single type of dysbiosis (83.2%), and the remaining 17 had vaginal coinfections (16.8%). The most common coinfections found in women were BV and AV (12/17), followed by BV and VC (3/17), AV and VC (1/17), and lastly, all studied vaginal infections (1/17).

**Table 1:** Identification of the main vaginal *Lactobacillus* species among the population set of the study realized by Salinas and colleagues (2020).

	*Lactobacillus iners* N (%)		*Lactobacillus jensenii* N (%)		*Lactobacillus acidophilus* N (%)		*Lactobacillus crispatus* N (%)		*Lactobacillus gasseri* N (%)		Total N (%)
	Absence	Presence	Absence	Presence	Absence	Presence	Absence	Presence	Absence	Presence	
Total incidence	162 (37.2)	274 (62.8)	310 (71.1)	126 (28.9)	314 (72.0)	122 (28.0)	391 (89.7)	45 (10.3)	316 (72.5)	120 (27.5)	436 (100.0)
**Vaginal microbiota^†^**											
Healthy microbiota	101 (23.2)	187 (42.9)	195 (44.7)	93 (21.3)	194 (44.5)	94 (21.6) *	258 (59.2)	30 (6.9)	199 (45.6)	89 (20.4) *	288 (66.1)
Intermediate microbiota	23 (5.3)	24 (5.5)	34 (7.8)	13 (3.0)	39 (8.9)	8 (1.8)	42 (9.6)	5 (1.1)	40 (9.2)	7 (1.6)	47 (10.8)
Bacterial vaginosis	10 (2.3)	14 (3.2)	19 (4.4)	5 (1.1)	21 (4.8)	3 (0.7)	23 (5.3)	1 (0.2)	20 (4.6)	4 (0.9)	24 (5.5)
Aerobic vaginitis	22 (5.0)	31 (7.1)	45 (10.3)	8 (1.8)	43 (9.9)	10 (2.3)	47 (10.8)	6 (1.4)	40 (9.2)	13 (3.0)	53 (12.2)
Candidiasis	0 (0.0)	7 (1.6)	5 (1.1)	2 (0.5)	4 (0.9)	3 (0.7)	6 (1.4)	1 (0.2)	3 (0.7)	4 (0.9)	7 (1.6)
Coinfections	6 (1.4)	11 (2.5)	12 (2.8)	5 (1.1)	13 (3.0)	4 (0.9)	15 (3.4)	2 (0.5)	14 (3.2)	3 (0.7)	17 (3.9)
**Age**											
≤21	59 (13.5)	109 (25.0)	115 (26.4)	53 (12.2)	126 (28.9)	42 (9.6)	156 (35.8)	12 (2.8)	125 (28.7)	43 (9.9)	168 (38.5)
22–28	53 (12.2)	112 (25.7)	118 (27.1)	47 (10.8)	109 (25.0)	56 (12.8) *	137 (31.4)	28 (6.4) **	118 (27.1)	47 (10.8)	165 (37.8)
29–35	16 (3.7)	18 (4.1)	27 (6.2)	7 (1.6)	22 (5.0)	12 (2.8)	30 (6.9)	4 (0.9)	25 (5.7)	9 (2.1)	34 (7.8)
36–42	12 (2.8)	14 (3.2)	17 (3.9)	9 (2.1)	19 (4.4)	7 (1.6)	25 (5.7)	1 (0.2)	16 (3.7)	10 (2.3)	26 (6.0)
43–49	3 (0.7)	6 (1.4)	5 (1.1)	4 (0.9)	7 (1.6)	2 (0.5)	9 (2.1)	0 (0.0)	6 (1.4)	3 (0.7)	9 (2.1)
≥50	5 (1.1)	7 (1.6)	9 (2.1)	3 (0.7)	9 (2.1)	3 (0.7)	12 (2.8)	0 (0.0)	8 (1.8)	4 (0.9)	12 (2.8)
Did not answer	14 (3.2)	8 (1.8)	19 (4.4)	3 (0.7)	22 (5.0)	0 (0.0)	22 (5.0)	0 (0.0)	18 (4.1)	4 (0.9)	22 (5.0)
**Ethnicity**											
Hispanic	134 (30.7)	251 (57.6) **	270 (61.9)	115 (26.4)	271 (62.2)	114 (26.1)	345 (79.1)	40 (9.2)	272 (62.4)	113 (25.9)	385 (88.3)
Indigenous	2 (0.5)	4 (0.9)	5 (1.6)	1 (0.2)	6 (1.4)	0 (0.0)	6 (1.4)	0 (0.0)	6 (1.4)	0 (0.0)	6 (1.4)
Caucasian	2 (0.5)	4 (0.9)	4 (0.9)	2 (0.5)	4 (0.9)	2 (0.5)	5 (1.1)	1 (0.2)	5 (1.1)	1 (0.2)	6 (1.4)
Afro-Ecuadorian	1 (0.2)	0 (0.0)	1 (0.2)	0 (0.0)	1 (0.2)	0 (0.0)	1 (0.2)	0 (0.0)	1 (0.2)	0 (0.0)	1 (0.2)
Did not answer	23 (5.3)	15 (3.4)	30 (6.9)	8 (1.8)	32 (7.3)	6 (1.4)	34 (7.8)	4 (0.9)	32 (7.3)	6 (1.4)	38 (8.7)
**Occupation**											
Housewife	1 (0.2)	6 (1.4)	4 (0.9)	3 (0.7)	6 (1.4)	1 (0.2)	7 (1.6)	0 (0.0)	5 (1.1)	2 (0.5)	7 (1.6)
Student	98 (22.5)	208 (47.7) **	210 (48.2)	96 (22.0)	216 (49.5)	90 (20.6)	273 (62.8)	33 (7.6) *	226 (51.8)	80 (18.3)	306 (70.2)
Unprofessional	12 (2.8)	13 (3.0)	21 (4.8)	4 (0.9)	19 (4.4)	6 (1.4)	25 (5.7)	0 (0.0)	15 (3.4)	10 (2.3)	25 (5.7)
Professional	36 (8.3)	38 (8.7)	55 (12.6)	19 (4.4)	50 (11.5)	24 (5.5)	62 (14.2)	12 (2.8)	50 (11.5)	24 (5.5)	74 (17.0)
Did not answer	15 (3.4)	9 (2.1)	20 (4.6)	4 (0.9)	23 (5.3)	1 (0.2)	24 (5.5)	0 (0.0)	20 (4.6)	4 (0.9)	24 (5.5)
**Civil status**											
Married	21 (4.8)	31 (7.1)	37 (8.5)	15 (3.4)	38 (8.7)	14 (3.2)	48 (11.0)	4 (0.9)	34 (7.8)	18 (4.1)	52 (11.9)
Divorced	4 (0.9)	5 (1.1)	7 (1.6)	2 (0.5)	8 (1.8)	1 (0.2)	9 (2.1)	0 (0.0)	5 (1.1)	4 (0.9)	9 (2.1)
Single with partner	51 (11.7)	132 (30.3) **	120 (27.5)	63 (14.4)	119 (27.3)	64 (14.7) **	156 (35.8)	27 (6.2) *	124 (28.4)	59 (13.5) *	183 (42.0)
Single without partner	67 (15.4)	93 (21.3)	121 (27.8)	39 (8.9)	122 (28.0)	38 (8.7)	148 (33.9)	12 (2.8)	128 (29.4)	32 (7.3)	160 (36.7)
Free union	4 (0.9)	4 (0.9)	5 (1.1)	3 (0.7)	4 (0.9)	4 (0.9)	6 (1.4)	2 (0.5)	5 (1.1)	3 (0.7)	8 (1.8)
Did not answer	15 (3.4)	9 (2.1)	20 (4.6)	4 (0.9)	23 (5.3)	1 (0.2)	24 (5.5)	0 (0.0)	20 (4.6)	4 (0.9)	24 (5.5)
**Sexual partner**											
With partner	77 (17.7)	169 (38.8) **	164 (37.6)	82 (18.8) *	163 (37.4)	83 (19.0) ***	213 (48.9)	33 (7.6) *	165 (37.8)	81 (18.6) **	246 (56.4)
Without partner	71 (16.3)	97 (22.2)	127 (29.1)	41 (9.4)	129 (29.6)	39 (8.9)	156 (35.8)	12 (2.8)	133 (30.5)	35 (8.0)	168 (38.5)
Did not answer	14 (3.2)	8 (1.8)	19 (4.4)	3 (0.7)	22 (5.0)	0 (0.0)	22 (5.0)	0 (0.0)	18 (4.1)	4 (0.9)	22 (5.0)
**Education level**											
≤Basic	2 (0.5)	4 (0.9)	6 (1.4)	0 (0.0)	5 (1.1)	1 (0.2)	6 (1.4)	0 (0.0)	5 (1.1)	1 (0.2)	6 (1.4)
Secondary	109 (25.0)	221 (50.7) **	230 (52.8)	100 (22.9)	233 (53.4)	97 (22.2) **	298 (68.3)	32 (7.3)	240 (55.0)	90 (20.6)	330 (75.7)
≥University	37 (8.5)	40 (9.2)	54 (12.4)	23 (5.3)	53 (12.2)	24 (5.5)	64 (14.7)	13 (3.0)	52 (11.9)	25 (5.7)	77 (17.7)
Did not answer	14 (3.2)	9 (2.1)	20 (4.6)	3 (0.7)	23 (5.3)	0 (0.0)	23 (5.3)	0 (0.0)	19 (4.4)	4 (0.9)	23 (5.3)
**Birth control methods**											
Condom	24 (5.5)	50 (11.5)	53 (12.2)	21 (4.8)	45 (10.3)	29 (6.7)	62 (14.2)	12 (2.8)	52 (1.9)	22 (5.0)	74 (17.0)
Other than condom	57 (13.1)	121 (27.8) **	121 (27.8)	57 (13.1)	123 (28.2)	55 (12.6) **	157 (36.0)	21 (4.8) **	121 (27.8)	57 (13.1)	178 (40.8)
None	62 (14.2)	90 (20.6)	112 (25.7)	40 (9.2)	118 (27.1)	34 (7.8)	145 (33.3)	7 (1.6)	118 (27.1)	34 (7.8)	152 (34.9)
Did not answer	19 (4.4)	13 (3.0)	24 (5.5)	8 (1.8)	28 (6.4)	4 (0.9)	27 (6.2)	5 (1.1)	25 (5.7)	7 (1.6)	32 (7.3)

N, number of women who responded in the survey within each category; %, assigned percentage for each classification within each category. The chi-square test was used to evaluate associations between the prevalence of each Lactobacillus sp. with the other risk factors. p < 0.05 and 95% CIs were considered significant for the test: *p ≤ 0.05; **p ≤ 0.02; ***p ≤ 0.001.

^†^Vaginal microbiota diagnoses, sociodemographic, and behavioral variables among the population set based on the previous study by Salinas et al. (2020).

Approximately 87.2% of the population set was constituted by undergraduate students or young professionals (380/436). The categories of professionals included the following: health professionals (23.0%), administrative clerks (20.3%), educators (14.9%), and general employees with college degrees (18.0%). The remaining professions without specialization or need for college degrees were classified as unprofessional careers. Most of the volunteers were single women (78.7%) and of Hispanic ethnicity (88.3%). Among the participants, 56.4% had a steady sexual partner, and 38.5% reported not having any sexual partner. Concerning birth control methods, 17.0% of participants reported using a condom, 40.8% reported the use of other birth control methods, and the remaining women did not use any birth control method (34.9%) or merely did not answer (7.3%). Alternative birth control methods included hormone treatment or other forms of protection (e.g., spermicides, diaphragm, cervical cap, and sterilization), intrauterine device (IUD), and natural methods (abstinence, fertility awareness method (FAM), and withdrawal). In our study, the most used alternative contraceptive method was hormonal, through oral contraceptives (46.7%) and local implants (6.6%).

**Figure 1:**
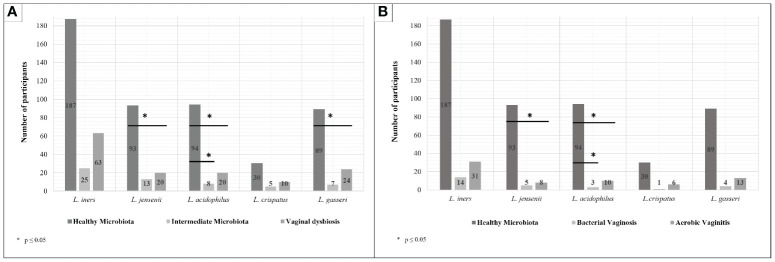
Prevalence of each *Lactobacillus* species according to the type of vaginal microbiota. **(A)** Lactobacilli prevalence in healthy microbiota, intermediate microbiota, and vaginal dysbiosis. **(B)** Lactobacilli prevalence in healthy microbiota, bacterial vaginosis (BV), and aerobic vaginitis (AV). Chi-square tests were performed among the prevalence of each *Lactobacillus* species in the presence of healthy microbiota, intermediate microbiota, or vaginal dysbiosis **(A)** and then BV and aerobic vaginitis (AV) **(B)**. **(A)** The results show statistically significant differences between healthy and intermediate microbiota in *Lactobacillus acidophilus* (*p* = 0.026) and *Lactobacillus gasseri* (*p* = 0.020). Meanwhile, statistically significant differences between healthy microbiota and vaginal dysbiosis were shown in presence of *Lactobacillus jensenii* (*p* = 0.017) and *L. acidophilus* (*p* = 0.015). However, no statistically significant differences were established between intermediate microbiota and vaginal dysbiosis. **(B)** The results show statistically significant differences between healthy microbiota and AV in *L. jensenii* (*p* = 0.012) and *L. acidophilus* (*p* = 0.045). Meanwhile, only statistically significant differences between healthy microbiota and BV were shown in presence of *L. acidophilus* (*p* = 0.041); no statistically significant differences were established between AV and BV.

When the results of the chi-square test in the prevalence of each *Lactobacillus* sp. between healthy microbiota, intermediate microbiota, and vaginal dysbiosis were analyzed, each group showed statistically significant differences in the presence of *L. acidophilus*, *L. jensenii*, and *L. gasseri*, as shown in **Figure 1A**. However, only *L. acidophilus* showed simultaneous statistical differences between healthy and intermediate microbiota (*p* = 0.026) and between healthy microbiota and vaginal dysbiosis (*p* = 0.015). The prevalence of both *L. jensenii* and *L. gasseri* was statistically different between healthy microbiota and vaginal dysbiosis (*p* = 0.017 and *p* = 0.020, respectively). No statistically significant differences were observed in these lactobacilli presence between intermediate microbiota and vaginal dysbiosis (see **Figure 1A**). Finally, *L. iners* and *L. crispatus* did not demonstrate statistical differences among these groups of the vaginal microbiota.

Further statistical analysis was then realized between healthy microbiota and specific types of vaginal dysbiosis (more exactly AV and BV), as well as between vaginal dysbioses. As shown in **Figure 1B**, some statistically significant differences were found on certain *Lactobacillus* species when comparing healthy microbiota against BV and AV, but no statistical differences were found between BV and AV. *L. acidophilus* showed statistically significant differences in its prevalence on healthy microbiota against both dysbioses (BV, *p* = 0.041; and AV, *p* = 0.045), while *L. jensenii* only showed statistically significant differences between healthy microbiota and AV cases (*p* = 0.012).

**Figure 2:**
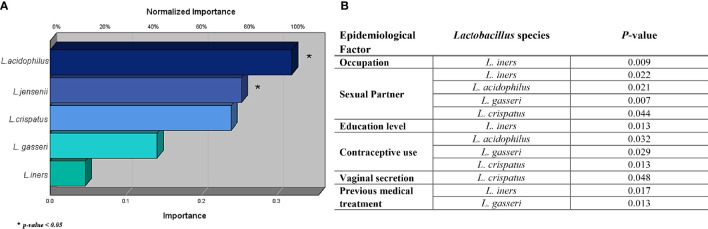
Protective effect of the identified *Lactobacillus* sp. against the development of vaginal dysbiosis **(A)** and their statistical significance with epidemiological factors **(B)** evaluated in the study. The effect of each *Lactobacillus* sp. against the presence of a vaginal dysbiosis case was evaluated using logistic regression, where *Lactobacillus acidophilus*, *Lactobacillus jensenii*, and *Lactobacillus crispatus* show the greatest importance. However, only *L. acidophilus* and *L. jensenii* featured a significant *p*-value, more exactly, 0.035 and 0.050, respectively. Meanwhile, *Lactobacillus gasseri* and *Lactobacillus iners* show a null protective effect against the infection when they are evaluated independently.

To evaluate if the statistically significant differences found in the prevalence of lactobacilli could have a protective effect against the development of vaginal dysbiosis, univariable logistic regression analyses were then performed. As shown in **Figure 2A**, each *Lactobacillus* species was normalized according to the importance of their presence against the vaginal dysbiosis establishment, showing *L. acidophilus*, *L. jensenii*, and *L. crispatus* importance of 100%, 79.3%, and 74.8%, respectively. However, only *L. acidophilus* and *L. jensenii* exhibited statistically significant differences (*p* = 0.035 and 0.050, respectively), suggesting a potential protective effect against the development of vaginal dysbiosis.

In addition, little is still known about epidemiological factors and lactobacilli colonization among women (Vaneechoutte, 2017a; Auriemma et al., 2021). Therefore, multiple chi-square analysis was also performed to evaluate possible correlations with each individual *Lactobacillus* species. As shown in **Figure 2B**, *L. iners* (*p* = 0.017) and *L. gasseri* (*p* = 0.013) were more prevalent in women with previous antimicrobial treatment in their clinical background. *L. crispatus* (*p* = 0.048) was associated with the presence of vaginal secretion among women. However, *L. iners* was also related to other epidemiological factors, such as occupation (*p* = 0.009) and education level (*p* = 0.013), showing statistical differences in its distribution among women in these categories. More exactly, a higher prevalence of *L. iners* was found in women with a secondary level of education (see **Table 1**). Finally, other factors, such as having a sexual partner and contraceptive use, demonstrated statistically significant values in relation to multiple *Lactobacillus* species (*L. acidophilus*, *L. gasseri*, *L. crispatus*, and *L. iners*) differing only in the absence of *L. iners* in contraceptive use. Although these results evaluated the species’ individual probiotic role in the vaginal microbiota, it is well known that a probiotic microbiota is characterized by a multi-microbial effect character and is not caused merely by an individual effect (Vaneechoutte, 2017b; Wieërs et al., 2020). Therefore, a multi-microbial analysis was performed to evaluate a potential symbiotic or antagonistic relationship between these *Lactobacillus* species against both cases of vaginal dysbiosis.

### Analysis of *Lactobacillus* Species Association by Clustering Model

**Figure 3:**
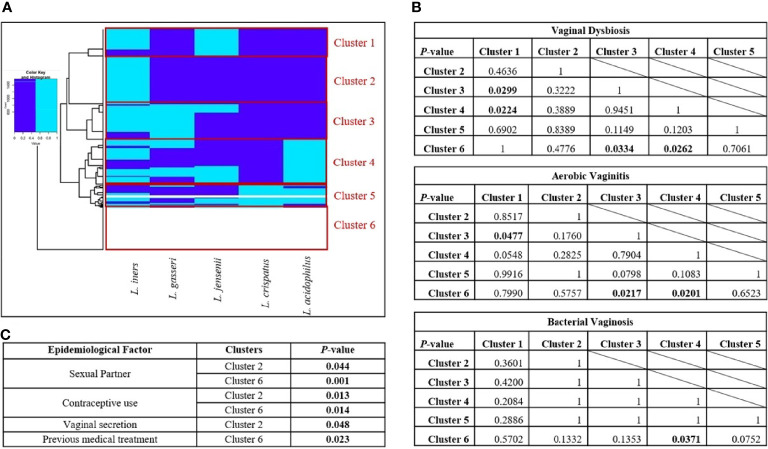
Clustering of the vaginal samples according to the prevalence of *Lactobacillus* sp. **(A)** Clusters obtained by Ward’s Minimum Variance Clustering Method. **(B)** Chi-square analysis between clusters in presence of vaginal dysbiosis, aerobic vaginitis, and bacterial vaginosis. **(C)** Epidemiological factors related to each cluster. Six clusters were chosen according to the presence of different *Lactobacillus* species in vaginal samples using Ward’s Minimum Variance Clustering Method. The following clusters are in panel **(A)**, *Cluster 1* was characterized by the presence of *Lactobacillus iners* and *Lactobacillus jensenii*; *Cluster 2* only showed *L. iners*; *Cluster 3* was constituted by *L. iners*, *L. jensenii*, and *Lactobacillus gasseri*; *Cluster 4* was formed by *L. iners*, *L. jensenii*, *L. gasseri*, and *Lactobacillus acidophilus*; *Cluster 5* is a mixture of all *Lactobacillus* species; and *Cluster 6* shows the absence of all of them. The dark blue color indicates the absence of a *Lactobacillus* species; meanwhile, the light blue color indicates the presence of the *Lactobacillus* species. In panel **(B)**, chi-square tests were performed to assess the statistical differences between clusters. The *p*-values where statistically significant differences were found are shown in bold. Finally, in panel **(C)**, multiple chi-square tests were performed to evaluate the epidemiological factors related to the presence of each cluster; the significant values are featured in the corresponding table in bold.

Nowadays, it is well known that the probiotic activity provided by a certain microbiota is caused not just by the effect of an individual *Lactobacillus* species but also by its multi-microbial interaction. So further analysis was also done through the clustering model of these *Lactobacillus* species against vaginal dysbiosis (by itself and then AV and BV) and epidemiological factors. A clustering model was realized through Ward’s Minimum Variance Clustering Method evidencing multiple clusters. The clustering of samples was developed according to the presence of different *Lactobacillus* species in vaginal samples. As shown in **Figure 3A**, six clusters were selected for multiple chi-square analysis to evaluate statistically significant differences (*p* ≤ 0.05). Cluster 1 was characterized by the presence of *L. iners* and *L. jensenii*, while Cluster 2 was only formed by *L. iners*. Cluster 3 was constituted by *L. iners*, *L. jensenii*, and *L. gasseri*. Cluster 4 assembled four *Lactobacillus* species, more exactly, *L. iners*, *L. jensenii*, *L. gasseri*, and *L. acidophilus*. Finally, Cluster 5 gathered all studied *Lactobacillus* species, and Cluster 6 evidenced no lactobacilli presence.

As shown in **Figure 3B**, only Clusters 3 and 4 demonstrated statistically significant differences against Clusters 1 and 6 in the establishment of vaginal dysbiosis. Clusters 3 and 4 shared *L. iners*, *L. jensenii*, and *L. gasseri*, but Cluster 4 also comprised *L acidophilus*. Both clusters evidenced a multi-species effect, being more notorious in Cluster 4 due to the obtained statistical values (*p* < 0.030). Interestingly, Cluster 1 also gathered *L. iners* and *L. jensenii* as Clusters 3 and 4; however, the absence of *L. gasseri* and *L. acidophilus* led to the lack of probiotic protection in vaginal dysbiosis establishment. The absence of *Lactobacillus* species in Cluster 6 was expected to relate to vaginal dysbiosis. It is also important to highlight that no statistical differences were found between Cluster 3 and 4 or even between Cluster 1 and 6, suggesting a potential probiotic connection among them. When individually evaluating each vaginal dysbiosis, greater statistically significant differences were found in the presence of AV (three *p*-values ≤0.05) than BV (one *p*-value ≤0.05). In AV cases, clustering analysis showed statistically significant differences in Clusters 3 and 4 when compared to Cluster 6 (*p* = 0.022 and *p* = 0.020, respectively), but only Cluster 3 showed statistical difference against Cluster 1 (*p* = 0.048). However, in BV cases, only Cluster 4 showed a statistically significant difference against Cluster 6 (*p* = 0.009), which is characterized by the absence of *Lactobacillus* species. In AV and BV cases, the combination of *L. iners*, *L. jensenii*, *L. gasseri*, and *L. acidophilus* from Cluster 4 reflected a multi-microbial consortium with statistical differences in the establishment of both dysbioses.

Finally, multiple chi-square analysis was also performed to evaluate the epidemiological factors related to the presence of each Cluster. As shown in **Figure 3C**, only Clusters 2 and 6 showed statistically significant differences among epidemiological factors. Both clusters shared statistically significant differences in women with a sexual partner and no contraceptive use (see **Figure 3C**), evidencing an association between these epidemiological behaviors and the lack of lactobacilli apart from *L. iners*. However, only Cluster 2 was associated with vaginal secretion in women (*p* = 0.048), more exactly, the presence of *L. iners* and the absence of the remaining analyzed lactobacilli, while Cluster 6 was correlated to women with a previous clinical history of antibiotic treatment for vaginal dysbiosis (*p* = 0.023), suggesting a potential correlation between treatments and eradication of vaginal lactobacilli.

### Association Between the Presence of *Lactobacillus* sp. and Opportunistic Pathogens

To determine the relationship between the presence of any opportunistic pathogens and each *Lactobacillus* species analyzed in this study or the clusters formed by them, multiple chi-square tests were realized (see **Supplementary Tables 2**, **3**). The opportunistic pathogens previously identified in our last study were *Gardnerella* spp., *F. vaginae* (previously known as *A. vaginae*) and *Mobiluncus* spp. related with BV, *E. coli* related with AV, and *C. albicans* related with candidiasis (Salinas et al., 2020). As shown in **Supplementary Table 2**, every opportunistic pathogen showed a statistical significance for at least one *Lactobacillus* species, except for *Gardnerella* genus.

Interestingly, the presence of *L. iners* was statistically correlated with the absence of *Mobiluncus* species (*p* = 0.033). On the contrary, the absence of *L. jensenii* was statistically associated with the absence of *C. albicans* (*p* = 0.034), while the absence of *L. acidophilus* evidenced the same association with *F. vaginae* (*p* < 0.001) and *E. coli* (*p* = 0.015). Meanwhile, *L. crispatus* showed multiple statistical associations with *F. vaginae*, *C. albicans* (both *p* < 0.001), and *E. coli* (*p* = 0.005), where its absence was correlated with the absence of these opportunistic microorganisms. Finally, no statistical correlation was found in the presence or absence of *L. gasseri* in the vaginal epithelium among Ecuadorian women.

To better understand the multispecies probiotic activity of lactobacilli, multiple chi-square tests were further studied in the lactobacilli clusters. The results showed significant values among clusters 1, 2, 4, and 6, as shown in **Supplementary Table 3**. As previously stated, these clusters represent the presence of *L. iners* and *L. jensenii* (Cluster 1), only *L. iners* (Cluster 2), all lactobacilli except for *L. crispatus* (Cluster 4), and the absence of *Lactobacillus* sp. (Cluster 6). As shown in **Supplementary Table 3**, Cluster 1 evidenced an inhibition of the presence of *Gardnerella* genus (*p* = 0.04). Cluster 2 is particularly interesting showing multiple statistical associations, illustrating a proliferation of *F. vaginae* (*p* = 0.001) and an inhibition of *C. albicans* (*p* = 0.001) and *E. coli* (*p* = 0.006). *Mobiluncus* spp. showed an opposite effect with the presence of Cluster 4, while the absence of *Lactobacillus* sp. in Cluster 6 could be inhibiting the proliferation of *F. vaginae* (*p* = 0.05).

## Discussion

The vaginal microbiota plays a vital role in modulating the risk of vaginal dysbiosis (Salinas et al., 2020). The protective role of *Lactobacillus* species in maintaining a healthy vaginal state in women is well known (Pacha-Herrera et al., 2020). However, this probiotic protection is caused not just by the individual effect of *Lactobacillus* species but also by its multi-microbial interaction (Graf et al., 2019). Little is still known about this multi-microbial dynamic among lactobacilli. Herein, we evaluated the individual and collective analyses of the prevalence of five lactobacilli (*L. iners*, *L. crispatus*, *L. gasseri*, *L. jensenii*, and *L. acidophilus*) among healthy women and women with vaginal dysbiosis, more exactly, BV and AV.

The chi-square, univariable, and multivariable logistic regression analyses with the BH adjustment allowed us to evaluate the possible associations between each *Lactobacillus* species and vaginal microbiota. According to the univariable logistic regression analysis for determining the protective effect against vaginal dysbiosis, *L. acidophilus*, *L. jensenii*, and *L. crispatus* demonstrated excellent normalized importance values of 100%, 79.3%, and 74.8%, respectively. Moreover, *L. acidophilus* and *L. jensenii* exhibited statistically significant values, more exactly, *p* = 0.035 and *p* = 0.050, respectively. However, only *L. acidophilus* showed statistically significant differences in its prevalence on healthy microbiota against both dysbioses (BV, *p* = 0.041; and AV, *p* = 0.045), whereas *L. jensenii* only showed statistically significant differences between healthy microbiota and AV cases (*p* = 0.012). Although these findings are in agreement with previous studies (Hütt et al., 2016; Chee et al., 2020), *L. acidophilus* evidenced a higher probiotic effect than the vaginal consortia previously described by Chee and colleagues, and both *L. acidophilus* and *L. jensenii* showed a significant probiotic effect against AV development, which was not previously reported, to the best of our knowledge.

Furthermore, the multi-microbial clustering model done by Ward’s Minimum Variance Clustering Method with Euclidean squared distance for hierarchical clustering allowed us to estimate the symbiotic relationship between these *Lactobacillus* species against both cases of vaginal dysbiosis. Our results evidenced a plausible strong probiotic multi-microbial consortium by *L. iners*, *L. jensenii*, *L. gasseri*, and *L. acidophilus* against AV (*p* = 0.020) and BV (*p* = 0.009). In addition, the absence of *L. gasseri* and *L. acidophilus* in other lactobacilli clusters leads to the lack of probiotic protection in vaginal dysbiosis establishment. These results are also in concordance with the predominance of bacterial consortia in our previous exploratory analysis among Ecuadorian teenagers against BV establishment (Salinas et al., 2018) and complementing information about lactobacilli combinations in probiotic formulas for the vaginal health against urogenital pathogens by Nader-Macías and colleagues (Nader-Macías et al., 2021).

Overall, this study shows the multi-microbial probiotic protection of these lactobacilli (*L. jensenii*, *L. gasseri*, and *L. acidophilus*) against both dysbioses. *L. jensenii* showed an individual probiotic effect against AV. Although the protective effect of *L. gasseri* against BV is well known (Scillato et al., 2021), its individual effect is overlapped when other *Lactobacillus* species are present in the same cluster, in particular *L. iners* and *L. crispatus*. However, the present study has several limitations, such as the absence of longitudinal analysis between vaginal infections and sociodemographic/behavioral variables or lactobacilli, only one vaginal sample was collected of each volunteer, and the lack of quantitative data. Therefore, the results of the present study could lead to an underestimation of the prevalence of opportunistic pathogens or even an overestimation of the probiotic activity of lactobacilli. Further studies should be conducted in Ecuador to quantify lactobacilli in different vaginal microbiota types verifying their probiotic activities among women.

## Data Availability Statement

The original contributions presented in the study are included in the article/**Supplementary Material**. Further inquiries can be directed to the corresponding author.

## Ethics Statement

The studies involving human participants were reviewed and approved by the Ethics Committee of the Universidad San Francisco de Quito (USFQ) (Protocol code: 2016-023IN by MSP-VGVS-2016-0244-O review board). The patients/participants provided their written informed consent to participate in this study.

## Author Contributions

Experimental research: DP-H, MPE-G, DC, MO, PB-S, and CA. Methodology: AM. Validation: DP-H, MPE-G, DC, MO, PB-S, and CA. Formal analysis: DP-H, ET, and AM. Resources: AM. Data curation: DP-H, ET, and AM. Writing—original draft preparation: DP-H and AM. Writing—review and editing: DP-H, ET, and AM. Supervision: AM. Project administration and funding: AM with Universidad San Francisco de Quito (USFQ) Chancellor Grants. All authors listed have made a substantial, direct, and intellectual contribution to the work and approved it for publication.

## Funding

This work is supported by Chancellor Grants 2018 and Colegio de Ciencias Biológicas y Ambientales research budget from Universidad San Francisco de Quito, under the Project ID: 5456 entitled “Caracterización de la microbiota vaginal y sus factores de riesgos en mujeres ecuatorianas.” The funders had no role in study design, data collection, analysis, decision to publish, or preparation of the manuscript.

## Conflict of Interest

The authors declare that the research was conducted in the absence of any commercial or financial relationships that could be construed as a potential conflict of interest.

## Publisher’s Note

All claims expressed in this article are solely those of the authors and do not necessarily represent those of their affiliated organizations, or those of the publisher, the editors and the reviewers. Any product that may be evaluated in this article, or claim that may be made by its manufacturer, is not guaranteed or endorsed by the publisher.

## Supplementary Material

Supplementary Table 1 PCR primers used in this study.

Supplementary Table 2 Evaluation of potential associations between the presence of *Lactobacillus* sp. and opportunistic pathogens. Multiple chi-square tests were performed to evaluate the absence or presence of each pathogen during the presence of each *Lactobacillus* sp., showing the *P*-values with statistically significant differences as bold values.

Supplementary Table 3 Evaluation of potential associations between opportunistic pathogens with clustering of *Lactobacillus* sp. Multiple chi-square tests were performed to evaluate the absence or presence of each pathogen during the presence of each cluster, showing the *P*-values with statistically significant differences as bold values.
