# Supplementary material for: Clustering Analysis of the Multi-Microbial Consortium by Lactobacillus Species Against Vaginal Dysbiosis Among Ecuadorian Women
Source: Front Cell Infect Microbiol. 2022 May 11;12:863208. doi: 10.3389/fcimb.2022.863208 (PMC9131875; doi:10.3389/fcimb.2022.863208)
Supplement: Supplementary Table 1 — PCR primers used in this study. [file Table_1.docx]

**Supplementary Table 1.** PCR primers used in this study.

| **Set** | **Primer Name** | **Sequence (5′-3′)** | **Target** | **T (°C) of annealing** | **Size of fragment** | **Reference** |
| --- | --- | --- | --- | --- | --- | --- |
| 1 | LacidoF | TGGAAACAGRTGCTAATACCG | *L. acidophillus* | 56 °C | 221 bp | (Tsai et al., 2010) |
|  | LacidoR | GTCCATTGTGGAAGATTCCC |  |  |  |  |
| 2 | LinersF | GTCTGCCTTGAAGATCGG | *L. iners* | 55 °C | 158 bp | (De Backer et al., 2007; Zhang et al., 2012) |
|  | LinersR | ACAGTTGATAGGCATCATC |  |  |  |  |
| 3 | LcrispatusF | TTACTTCGGTAATGACGTTA | *L. crispatus* | 55 °C | 966 bp | (De Backer et al., 2007; Zhang et al., 2012) |
|  | LcrispatusR | GGAACTTTGTATCTCTACAA |  |  |  |  |
| 4 | LgasseriF | TCGAGCGAGCTTGCCTAGATGAA | *L. gasseri* | 60 °C | 372 bp | (Zhang et al., 2012) |
|  | LgasseriR | CGCGGCGTTGCTCCATCAGA |  |  |  |  |
| 5 | LjenseniiF | AGTCGAGCGAGCTTGCCTATAGAAG | *L. jensenii* | 57 °C | 342 bp | (Garg et al., 2009; Zhang et al., 2012) |
|  | LactoR | CTCACCCATCCGCCGCTAGCT |  |  |  |  |
