# Supplementary material for: Clustering Analysis of the Multi-Microbial Consortium by Lactobacillus Species Against Vaginal Dysbiosis Among Ecuadorian Women
Source: Front Cell Infect Microbiol. 2022 May 11;12:863208. doi: 10.3389/fcimb.2022.863208 (PMC9131875; doi:10.3389/fcimb.2022.863208)
Supplement: Supplementary Table 2 — Evaluation of potential associations between the presence of Lactobacillus sp. and opportunistic pathogens. Multiple chi-square tests were performed to evaluate the absence or presence of each pathogen during the presence of each Lactobacillus sp., showing the P-values with statistically significant differences as bold values. [file Table_2.docx]

**Supplementary Table 2.** Evaluation of potential associations between the presence of *Lactobacillus* sp. and opportunistic pathogens.

|  |  | **Gardnerella spp.** | | **F. vaginae** | | **C. albicans** | | **E. coli** | | **Mobiluncus spp.** | |
| --- | --- | --- | --- | --- | --- | --- | --- | --- | --- | --- | --- |
| **Lactobacillus species** | | **Absence** | **Presence** | **Absence** | **Presence** | **Absence** | **Presence** | **Absence** | **Presence** | **Absence** | **Presence** |
| **L. iners** | **Absence** | 99 | 63 | 99 | 63 | 161 | 1 | 135 | 27 | 153 | 9 |
|  | **Presence** | 162 | 112 | 150 | 124 | 267 | 7 | 243 | 31 | 269 | 5 |
|  | ***P*-value** | 0.683 | | 0.194 | | 0.145 | | 0.112 | | **0.033** | |
| **L. jensenii** | **Absence** | 186 | 124 | 183 | 127 | 307 | 3 | 270 | 40 | 299 | 11 |
|  | **Presence** | 75 | 51 | 66 | 60 | 121 | 5 | 108 | 18 | 123 | 3 |
|  | ***P*-value** | 0.927 | | 0.203 | | **0.034** | | 0.700 | | 0.531 | |
| **L. acidophilus** | **Absence** | 195 | 119 | 196 | 118 | 310 | 4 | 280 | 34 | 306 | 8 |
|  | **Presence** | 66 | 56 | 53 | 69 | 118 | 4 | 98 | 24 | 116 | 6 |
|  | ***P*-value** | 0.126 | | **0.000** | | 0.161 | | **0.015** | | 0.208 | |
| **L. crispatus** | **Absence** | 238 | 153 | 244 | 147 | 389 | 2 | 345 | 46 | 377 | 14 |
|  | **Presence** | 23 | 22 | 5 | 40 | 39 | 6 | 33 | 12 | 45 | 0 |
|  | ***P*-value** | 0.206 | | **0.000** | | **0.000** | | **0.005** | | 0.197 | |
| **L. gasseri** | **Absence** | 196 | 120 | 180 | 136 | 312 | 4 | 271 | 45 | 306 | 10 |
|  | **Presence** | 65 | 55 | 69 | 51 | 116 | 4 | 107 | 13 | 116 | 4 |
|  | ***P*-value** | 0.135 | | 0.919 | | 0.151 | | 0.349 | | 0.929 | |

Legend: Multiple chi-square tests were performed to evaluate the absence or presence of each pathogen during the presence of each *Lactobacillus* sp., showing the *P*-values with statistically significant differences as bold values.
