# Supplementary material for: Clustering Analysis of the Multi-Microbial Consortium by Lactobacillus Species Against Vaginal Dysbiosis Among Ecuadorian Women
Source: Front Cell Infect Microbiol. 2022 May 11;12:863208. doi: 10.3389/fcimb.2022.863208 (PMC9131875; doi:10.3389/fcimb.2022.863208)
Supplement: Supplementary Table 3 — Evaluation of potential associations between opportunistic pathogens with clustering of Lactobacillus sp. Multiple chi-square tests were performed to evaluate the absence or presence of each pathogen during the presence of each cluster, showing the P-values with statistically significant differences as bold values. [file Table_3.docx]

**Supplementary Table 3.** Evaluation of potential associations between opportunistic pathogens with clustering of *Lactobacillus* sp.

|  |  | **Gardnerella spp.** | | **F. vaginae** | | **C. albicans** | | **E. coli** | | **Mobiluncus spp.** | |
| --- | --- | --- | --- | --- | --- | --- | --- | --- | --- | --- | --- |
| **Cluster** | | **Absence** | **Presence** | **Absence** | **Presence** | **Absence** | **Presence** | **Absence** | **Presence** | **Absence** | **Presence** |
| **1** | **Absence** | 197 | 146 | 196 | 147 | 335 | 8 | 299 | 44 | 330 | 13 |
|  | **Presence** | 65 | 29 | 54 | 40 | 94 | 0 | 79 | 15 | 93 | 1 |
|  | ***P*-value** | **0.040** | | 0.958 | | 0.135 | | 0.432 | | 0.184 | |
| **2** | **Absence** | 239 | 153 | 245 | 147 | 390 | 2 | 345 | 47 | 378 | 14 |
|  | **Presence** | 23 | 22 | 5 | 40 | 39 | 6 | 33 | 12 | 45 | 0 |
|  | ***P*-value** | 0.201 | | **0.001** | | **0.001** | | **0.006** | | 0.198 | |
| **3** | **Absence** | 228 | 154 | 212 | 170 | 374 | 8 | 328 | 54 | 369 | 13 |
|  | **Presence** | 34 | 21 | 38 | 17 | 55 | 0 | 50 | 5 | 54 | 1 |
|  | ***P*-value** | 0.763 | | 0.057 | | 0.279 | | 0.306 | | 0.533 | |
| **4** | **Absence** | 212 | 136 | 197 | 151 | 340 | 8 | 301 | 47 | 340 | 8 |
|  | **Presence** | 50 | 39 | 53 | 36 | 89 | 0 | 77 | 12 | 83 | 6 |
|  | ***P*-value** | 0.415 | | 0.617 | | 0.149 | | 0.996 | | **0.034** | |
| **5** | **Absence** | 221 | 146 | 206 | 161 | 361 | 6 | 313 | 54 | 355 | 12 |
|  | **Presence** | 41 | 29 | 44 | 26 | 68 | 2 | 65 | 5 | 68 | 2 |
|  | ***P*-value** | 0.797 | | 0.297 | | 0.485 | | 0.089 | | 0.857 | |
| **6** | **Absence** | 213 | 140 | 194 | 159 | 345 | 8 | 304 | 49 | 343 | 10 |
|  | **Presence** | 49 | 35 | 56 | 28 | 84 | 0 | 74 | 10 | 80 | 4 |
|  | ***P*-value** | 0.763 | | **0.050** | | 0.164 | | 0.634 | | 0.367 | |

Legend: Multiple chi-square tests were performed to evaluate the absence or presence of each pathogen during the presence of each cluster, showing the *P*-values with statistically significant differences as bold values.
